# Myeloablative Conditioning with PBSC Grafts for T Cell-Replete Haploidentical Donor Transplantation Using Posttransplant Cyclophosphamide

**DOI:** 10.1155/2016/9736564

**Published:** 2016-01-21

**Authors:** Scott R. Solomon, Melhem Solh, Lawrence E. Morris, H. Kent Holland, Asad Bashey

**Affiliations:** Blood and Marrow Transplant Program at Northside Hospital, Atlanta, GA 30342, USA

## Abstract

Relapse is the main cause of treatment failure after nonmyeloablative haploidentical transplant (haplo-HSCT). In an attempt to reduce relapse, we have developed a myeloablative (MA) haplo-HSCT approach utilizing posttransplant cyclophosphamide (PT/Cy) and peripheral blood stem cells as the stem cell source. We summarize the results of two consecutive clinical trials, using a busulfan-based (*n* = 20) and a TBI-based MA preparative regimen (*n* = 30), and analyze a larger cohort of 64 patients receiving MA haplo-HSCT. All patients have engrafted with full donor chimerism and no late graft failures. Grade III-IV acute GVHD and moderate-severe chronic GVHD occurred in 23% and 30%, respectively. One-year NRM was 10%. Predicted three-year overall survival, disease-free survival, and relapse were 53%, 53%, and 26%, respectively, in all patients and 79%, 74%, and 9%, respectively, in patients with a low/intermediate disease risk index (DRI). In multivariate analysis, DRI was the most significant predictor of survival and relapse. Use of TBI (versus busulfan) had no significant impact on survival but was associated with significantly less BK virus-associated hemorrhagic cystitis. We contrast our results with other published reports of MA haplo-HSCT PT/Cy in the literature and attempt to define the comparative utility of MA haplo-HSCT to other methods of transplantation.

## 1. Introduction

Seventy percent of patients who urgently need an allogeneic hematopoietic stem cell transplantation (HSCT) do not have an available HLA-matched sibling donor. In such patients, a search for an HLA-matched unrelated donor (MUD) can identify an 8/8 HLA-identical donor for approximately 30% to 40% of transplant recipients. The probability of finding an acceptable MUD varies by ethnic groups, ranging from 75% in the white Europeans, to 30% to 40% in the Mexican and Central/South Americans, to 15% to 20% for the African Americans and black Caribbeans [1]. In addition, MUD transplantation is also complicated by the amount of time it takes from search initiation to transplantation, causing some patients to relapse or physically deteriorate while waiting for transplantation. In contrast, a haploidentical family member (haplo) can be identified and rapidly utilized in nearly all cases.

Historically, HSCT from a partially HLA-mismatched relative has been complicated by unacceptably high incidences of graft rejection, severe graft-versus-host disease (GVHD), and nonrelapse mortality (NRM) [2, 3]. To address the risk of graft rejection and GVHD, extensive T cell depletion has been utilized in association with antithymocyte globulin (ATG) and high peripheral blood stem cell (PBSC) dose [4]; however, NRM from infectious complications remains a challenge. More recently, the investigators at Johns Hopkins University have pioneered a method to selectively deplete alloreactive cells in vivo by administering high doses of cyclophosphamide (Cy) in a narrow window after transplantation [5]. After nonmyeloablative (NMA) conditioning, this approach has resulted in low NRM (4% and 15% at 1 and 2 years, resp.), because of low rates of GVHD and infectious complications. Immune reconstitution was promising with low risk of cytomegalovirus (CMV) or invasive mold infections. Using high-dose, posttransplantation cyclophosphamide (PT/Cy), crossing the HLA barrier in HSCT is now feasible without the need for extensive T cell depletion or serotherapy.

Studies of NMA haplo-HSCT with PT/Cy show remarkable tolerability of this approach with low rates of GVHD, infection, and NRM. Relapse of malignancy remains the predominant cause of treatment failure, occurring in approximately 45% to 51% of patients [5, 6]. NMA haplo-HSCT with PT/Cy has also been associated with an approximately 10% rate of engraftment failure resulting in autologous recovery. The use of more intense/myeloablative (MA) preparative regimens and PBSC grafts may potentially reduce the rate of relapse and graft rejection following haplo-HSCT PT/Cy transplants. However, only a limited number of such studies have been reported. In this paper, we report our experience with MA conditioning and PBSC allografts for T-replete haplo-HSCT using PT/Cy. We define the major predictors of outcome following this strategy. We also describe other published reports of MA haplo-HSCT PT/Cy in the literature. Finally, we compare the outcomes of MA and NMA haplo-HSCT using PT/Cy and attempt to define the comparative utility of MA haplo-HSCT in relation to MUD transplantation.

## 2. Busulfan-Based MA Haplo-HSCT (NSH 864 Protocol)

In a proof-of-principle study of MA haplo-HSCT, twenty patients with high risk hematologic malignancies were treated with a preparative regimen of fludarabine (125–180 mg/m^2^), i.v. busulfan (440–520 mg/m^2^) and Cy (29 mg/kg) before transplant, a G-CSF-mobilized PBSC graft, and posttransplant GVHD prophylaxis comprised of Cy 50 mg/kg/d on d +3 and +4, MMF 15 mg/kg three times daily d +5–+35, and tacrolimus (target 5–15 ng/mL) days +5 to +180 [7]. The median age of patients was 44 years (range: 25–56 years). Eleven patients (55%) underwent HSCT with relapsed/refractory disease (acute myelogenous leukemia [AML] 5, chronic myelogenous leukemia-blast crisis [CML-BC] 1, acute lymphoblastic leukemia 2, non-Hodgkin lymphoma 1, Hodgkin's disease 1, and chronic lymphocytic leukemia/Richters 1). The remaining patients had either AML CR1 with poor-risk cytogenetics and/or induction failure or chronic myelogenous leukemia resistant to all tyrosine kinase inhibitors.

All patients engrafted and demonstrated 100% donor chimerism in both peripheral blood T cell and myeloid cells from day +30. Cumulative incidence of one-year NRM was 10% and that of grade III-IV acute GVHD and severe chronic GVHD was 10% and 5%, respectively. Relapse was acceptable, occurring in 40% of patients, despite the fact that the majority had relapsed/refractory disease at time of transplant. With a median follow-up of 20 months, estimated probabilities of overall and disease-free survival (DFS) were 69% and 50%, respectively.

There were no cases of invasive mold infections or EBV-related PTLD. Only one patient had CMV disease and only one patient died of a viral infection (parainfluenza 3) suggesting that anti-infection immunity was preserved with this approach. However, nonfatal BK virus-associated hemorrhagic cystitis (HC) was seen in 75% of patients at a median of 38 days after transplant. Although it is not a life-threatening complication, it was a source of significant morbidity for some patients. We hypothesized that HC was predisposed to by the combined effect of high-dose busulfan and PT/Cy.

## 3. Total-Body Irradiation-Based Haplo-HSCT (NSH 922 Protocol)

In an attempt to reduce the risk of BK virus-associated HC, thirty patients were enrolled on prospective phase II trial utilizing a TBI-based myeloablative preparative regimen (fludarabine 25 mg/m^2^/d × 3 d and TBI 150 cGy bid on d −4 to −1 [total dose 1200 cGy]) followed by infusion of unmanipulated peripheral blood stem cells from a haploidentical family donor [8]. Postgrafting immunosuppression again consisted of Cy 50 mg/kg/day on days 3 and 4, MMF through d 35, and tacrolimus through d 180. Median patient age was 46.5 years (range 24–60). Transplant diagnosis included AML [9], ALL [6], CML [5], MDS [1], and NHL [2]. Using the revised Dana-Farber/CIBMTR disease risk index (DRI), patients were classified as having low [4], intermediate [10], high [11], and very high [3] risk.

**Figure 1:**
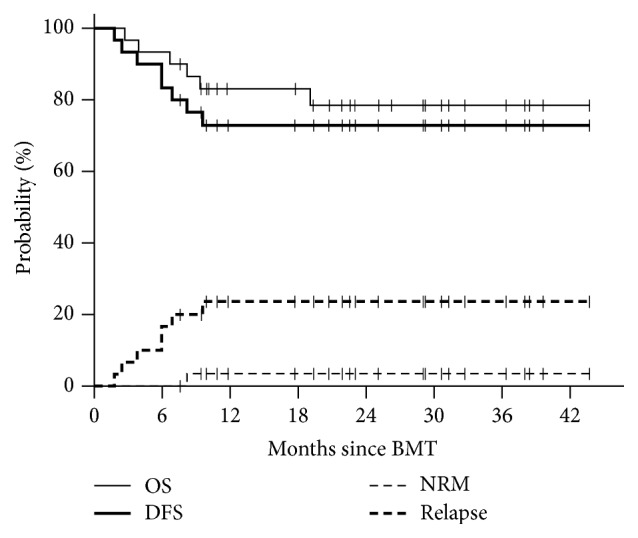
Kaplan-Meier analysis of overall survival, disease-free survival, and nonrelapse mortality and following TBI-based MA haplo-HSCT.

All patients engrafted with a median time to neutrophil and platelet recovery of 16 and 25 days, respectively. All evaluable patients achieved sustained complete donor T cell and myeloid chimerism by day +30. Acute GVHD, grades II–IV and III-IV, was seen in 43% and 23%, respectively. The cumulative incidence of moderate-to-severe chronic GVHD was 22% (severe in 10%). Nonrelapse mortality (NRM) at 2 years was 3%, which consisted of one death due to noninfectious respiratory failure/ARDS 8 months after transplant in a patient with chronic GVHD. Estimated two-year survival, DFS, and relapse were 78%, 73%, and 24%, respectively (Figure 1). Two-year DFS and relapse rate in patients with low/intermediate disease risk, determined by the DRI, were 100% and 0%, respectively, compared with 39% and 53% for patients with high/very high risk disease.

As noted in our prior experience with busulfan-based MA haplo-HSCT, posttransplant fever was common and occurred in the first 5 posttransplant days in nearly all patients. Fevers resolved in all patients following administration of PT/Cy. CMV reactivation (≥400 copies/mL) occurred in 15/26 (58%) of at-risk patients (either donor or recipient with CMV positive serostatus) at a median of day +43 after transplant (range 11–157). CMV disease did not occur. There were no episodes of invasive mold infection or infectious death in the first 100 days after transplant. There were no cases of EBV reactivation. BK virus-associated HC of any grade occurred in 30% of patients and was severe (grade ≥ 3) in 7%. As compared with our previous experience with busulfan-based MA haplo-HSCT, HC occurred significantly less often following TBI-based MA haplo-HSCT (any grade: 30% versus 75%, *p* = 0.005; severe HC: 7% versus 30%, *p* = 0.037).

## 4. Predictors of Outcome following MA Haplo-HSCT and PT/Cy

In order to determine predictors of outcome following MA haplo-HSCT and PT/Cy, we evaluated that sixty-four consecutive patients have been transplanted following either busulfan-based (*n* = 20; NSH 864) or TBI-based (*n* = 44; including 30 patients on NSH 922 and the remaining 14 patients treated identically after completion of the trial) MA conditioning, T cell-replete PBSC infusion, PT/Cy, and tacrolimus/mycophenolate mofetil. Median age of the cohort was 43 years (range 21–60). Patient characteristics included a high/very high disease risk by the Dana-Farber/CIBMTR disease risk index (DRI) in 32 patients (50%), KPS <90 in 69%, and comorbidity index (CMI) of ≥2 in 58% of patients. The most common indications for transplant were AML, ALL, and advanced-phase CML in 55%, 20%, and 12% of patients, respectively. Median follow-up for surviving patients was 24 months.

**Figure 2:**
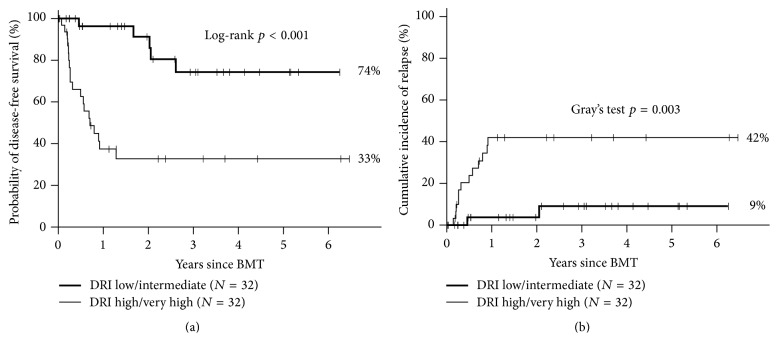
Effect of disease risk index on (a) disease-free survival and (b) relapse following MA haplo-HSCT.

All patients engrafted with full donor chimerism and no late graft failures. Grade II–IV, III-IV acute GVHD and moderate-severe chronic GVHD occurred in 46%, 23%, and 30%, respectively. One-year NRM was 10%. Predicted three-year overall survival (OS), disease-free survival (DFS), and relapse are 53%, 53%, and 26%, respectively. In the 32 patients with standard risk disease (low/intermediate DRI), outcomes were significantly improved with one-year NRM of 0% and predicted 3-year OS, DFS, and relapse of 79%, 74%, and 9%, respectively (Figure 2).

In multivariate analysis, high/very high DRI was the most significant negative predictor of OS (HR 13.26, *p* < 0.001), followed by CMI ≥2 (HR 3.54, *p* = 0.01) and age (HR 1.26, *p* = 0.038, per 5-year increase in age). DRI was also significantly associated with DFS (HR 10.84, *p* < 0.001), NRM (HR 15.0, *p* = 0.004), and relapse (HR 8.85, *p* = 0.004) (Table 1). Conditioning regimen (TBI versus busulfan) had no significant impact on OS, DFS, NRM, or relapse.

## 5. Additional Published Experience with MA Haplo-HSCT and PT/Cy

Several other groups have published similar experiences with MA haplo-HSCT with PT/Cy. Grosso et al. [12] reported a “two-step” strategy where a defined dose of haploidentical T cells (2 × 108/kg) was infused after MA doses of TBI. Patients then received 60 mg/kg of CY on two consecutive days, followed later by infusion of highly purified CD34+ cells from the donor. All patients engrafted and the cumulative incidence of grade III-IV acute GVHD and NRM was 7.4% and 22.5%, respectively, for the 27 patients treated. With a median follow-up of 40 months, overall survival was 48%. A second study from the same group [13], which included only patients in remission at the time of transplant, demonstrated a 2 yr NRM, relapse, and PFS of 4%, 19%, and 74%, respectively. The requirement for stringent ex vivo T depletion of the hematopoietic cell product differentiates this approach and may limit its widespread applicability. Furthermore, given the resistance of hematopoietic stem cells to Cy, such delayed infusion of selected CD34+ cells may be unnecessary.

Symons et al. [11] reported on 97 patients with either leukemias in complete remission or lymphoma with chemosensitive disease. Patients received MA haplo-HSCT PT/Cy utilizing bone marrow grafts. The preparative regimen consisted of IV busulfan (pharmacokinetically adjusted) on days −6 to −3 and Cy (50 mg/kg/day) on days −2 and −1, except for patients with acute lymphocytic leukemia or lymphoblastic lymphoma who received Cy (50 mg/kg/day) on days −5 and −4 and TBI (200 cGy twice daily) on days −3 to −1. Donor engraftment occurred in 73/82 (89%) patients. Estimated probabilities of NRM and grade III-IV acute GVHD at 100 days were 11% and 7%, respectively. The cumulative incidence of relapse was 44%. With a median follow-up of surviving patients of 474 days, estimated 2 yr overall and disease-free survival is 57% and 49%, respectively.

Raiola et al. [10] reported on 50 patients receiving a MA haplo-HSCT PT/Cy utilizing bone marrow grafts. The regimens used were thiotepa, busulfan, and fludarabine (*n* = 35) or TBI and fludarabine (*n* = 15). Forty-five patients (90%) engrafted with an 18-month cumulative incidence of NRM, relapse, and PFS of 18%, 22%, and 51%, respectively. PFS was 67% for patients transplanted in remission versus 37% for patients with active disease. Reported incidences of acute and chronic GVHD were low. As in our experience, HC was more common in patients receiving busulfan rather than TBI-based conditioning.

Whether PBSC or BM is the preferred stem cell source following MA haplo-HSCT remains unclear; however BM appears to be associated with a higher rate of graft failure, occurring in approximately 10% of patients in both the series by Raiola et al. [10] and the experience of Symons et al. [11]. Graft failure has not been reported with PBSC based myeloablative haplo-HSCT and PT/Cy.

## 6. Comparison of MA and NMA Haplo-PT/Cy

The overall risk of relapse associated with MA haplo-HSCT in the majority of studies is 20–25% [7, 8, 10, 13] and compares favorably with that reported for NMA haplo-HSCT (45–51%) [5, 6]. In our analysis of 64 patients receiving MA haplo-HSCT, relapse risk in patients with low (*n* = 7) or intermediate (*n* = 25) DRI was 9%, compared with 42% relapse rate in high (*n* = 24) or very high (*n* = 8) DRI patients. This compares favorably to that seen in the NMA setting, where relapse risk according to DRI was recently analyzed in 372 consecutive patients by the group from Johns Hopkins University [14]. In this analysis, the risk of relapse was also highly correlated with DRI, with relapse occurring in approximately 75%, 50%, and 20% of patients in the high/very high, intermediate, and low DRI groups, respectively. The finding of higher relapse following NMA conditioning parallels what has been seen following matched related or unrelated donor transplantation [9, 15–17].

## 7. Comparison of MA Haplo-PT/Cy with MA MUD Transplants

In order to evaluate the comparative efficacy of MA haplo-HSCT, we have compared outcomes of patients receiving TBI-based MA haplo-HSCT with PT/Cy (*n* = 30) with a contemporaneously treated cohort of consecutive patients at our institution receiving HLA-matched (8/8 HLA-A, HLA-B, HLA-C, and HLA-DR) MA T cell-replete MUD transplantation (*n* = 48) [8]. Haplo- and MUD transplant patients were well matched according to age, diagnosis, disease risk, CMV serostatus, and comorbidity index. The groups did differ in the use of PBSC as the stem cell source which was utilized in all haplotransplant recipients compared with 32 of 48 MUD transplants recipients. When compared with recipients of MA MUD transplants, outcomes after MA haplo-HSCT were statistically similar to 2 yr OS and DFS being 78% and 73%, respectively, after haplotransplant versus 71% and 64%, respectively, after MUD transplants. Grade II–IV acute GVHD was seen less often following haplotransplantation compared with MUD transplantation (43% versus 63%, *p* = 0.049), as was moderate-to-severe chronic GVHD (22% versus 58%, *p* = 0.003). The lower incidence of chronic GVHD occurred despite the greater use of PBSC in the haplo-HSCT group.

Similarly, a Center for International Blood and Marrow Transplant Research (CIBMTR) analysis [18] compared outcomes of adults with acute myeloid leukemia (AML) after haplo- (*n* = 192) and MUD (*n* = 1982) transplantation, including 104 MA haplotransplants and 1245 MA MUD transplants. In this large analysis, there were no significant differences in 1 yr NRM (12% versus 14%), 3 yr relapse (44% versus 39%), or 3 yr OS (46% versus 44%), comparing MA haplo- and MA MUD transplants, respectively. Grade II–IV acute GVHD (16% versus 33%), grade III-IV acute GVHD (7% versus 13%), and chronic GVHD (30% versus 53%) were all statistically lower in haplopatients compared with MUD patients.

## 8. Immune Recovery following MA Haplo-PT/Cy

Historically, MA haplotransplantation has been associated with considerable infectious morbidity and mortality. In contrast, our experience and others suggest that MA haplo-PT/Cy may significantly reduce the risk of infectious complications. In a published series of thirty patients undergoing TBI-based MA haplo-PT/Cy [8], CMV reactivation (≥400 copies/mL) occurred in only 15/26 (58%) of at-risk patients (either donor or recipient with CMV positive serostatus), and CMV disease did not occur. There were no episodes of invasive mold infection or infectious death in the first 100 days after transplant. Furthermore, there were no cases of EBV, HHV6, or adenovirus infections.

The reduced risk of infectious complications following MA haplo-PT/Cy has translated into low NRM, approximately 10% in the first year after transplant. Our experience compares favorably to the results reported with T cell-depleted (TCD) MA haplo, where NRM of approximately 40% have been seen, with much of this attributable to infectious mortality [4, 19–21]. Ciurea and colleagues at the MD Anderson Cancer Center analyzed their outcomes following MA haplo-PT/Cy following a preparative regimen of fludarabine, melphalan, and thiotepa, with historical results of TCD MA haplo using the same preparative regimen [20]. In this analysis, one-year NRM favored PT/Cy (16% versus 42%) as did death directly attributable to infection (9% versus 24%), with significantly less viral and fungal infections seen in PT/Cy versus TCD patients. T cell subset analysis demonstrated significant improvements in T cell recovery in PT/Cy versus TCD patients, with more rapid reconstitution noted in multiple T cell subsets (CD4, CD8, naïve, and memory).

Immune reconstitution following haplo-PT/Cy is characterized by a diverse T cell receptor repertoire and appears dependent on T memory stem cells maturing from naïve T cells [22, 23]. These cells are adoptively transferred in the donor graft and have been shown to survive cyclophosphamide-induced deletion. Furthermore, regulatory T cells also are preferentially preserved following PT/Cy, likely due to higher aldehyde dehydrogenase in these cells [24]. Finally, murine studies have demonstrated that PT/Cy relatively spares pathogen and cancer-specific T cells [25]. The selective elimination of alloreactive donor T cells with relative preservation of nonalloreactive donor T cell clones provides a mechanistic understanding of the surprisingly low infectious mortality following MA haplo-PT/Cy.

## 9. Discussion

In the past decade, there has been a growing interest in the use of haplo-HSCT due to the rapid and nearly universal availability of donors, which is a critical issue in patients with advanced hematologic malignancies. A major advance in the success of haplo-HSCT is the use of properly timed PT/Cy, a technique pioneered by investigators at Johns Hopkins University [5, 26]. Using a NMA approach, this strategy has resulted in low rates of GVHD, infection, and NRM. However, relapse remains the major cause of treatment failure, occurring in approximately half of transplant recipients. One explanation for the high rate of relapse, as in other NMA HSCT trials, is that the transplantation conditioning was not intense enough to achieve sufficient tumor cytoreduction.

In order to reduce the risk of relapse in patients with high risk hematologic malignancies, our group and others have demonstrated the feasibility of performing MA haplo-HSCT utilizing PT/Cy. In 64 consecutive patients transplanted at our institution following either busulfan-based (*n* = 20) or TBI-based (*n* = 44) MA conditioning, we have noted universal engraftment with rapid donor chimerism, acceptable rates of GVHD (grade III-IV acute GVHD and moderate-severe chronic GVHD occurred in 23% and 30%, resp.), and a low one-year NRM of 10%. Predicted three-year overall survival (OS), disease-free survival (DFS), and relapse were 53%, 53%, and 26%, respectively, and in the 32 patients with standard risk disease (low/intermediate DRI), outcomes were very favorable (3-year OS, DFS, and relapse of 79%, 74%, and 9%, resp.).

Relapse appears less following MA conditioning with relapse rates in the majority of studies of 20–25% [7, 8, 10, 13], compared with that reported for NMA haplo-BMT (45–51%) [5, 6]. However, truly defining the influence of the preparative regimen intensity on relapse risk will likely require a randomized controlled trial. When comparing our results with the other published experiences of MA haplo-HSCT using PT/Cy, it becomes evident that disease risk, as defined by either the DRI or disease status at the time of transplant, is the primary driver of outcomes, with 2 yr DFS being approximately 67–74% [8, 10, 13] in patients transplanted in remission without high risk disease defined by the DRI. Whether PBSC or BM is the preferred stem cell source following myeloablative haplo-HSCT remains unclear; however BM appears to be associated with a higher rate of graft failure, occurring in approximately 10% of patients [10, 11] receiving marrow grafts, and is obviously more consequential following MA conditioning.

Although there have been no randomized studies to date, there is now compelling evidence regarding the equivalent efficacy and safety of haplo-HSCT PT/Cy and MUD transplantation, in both the NMA and MA setting [8, 18, 27–29]. When considering the optimal transplant donor type, MUD versus haplo-HSCT, one must consider the inherent advantages of haplodonors including near universal and rapid availability, as well as lower costs related to donor searching and graft acquisition, whereas as almost all patients have an available haplomatched family member, the availability of an 8/8 matched unrelated donor varies according to ethnic background, ranging from 75% for white patients of European descent to less than 20% for the African Americans. Furthermore, given the complexities inherent in registry searching, time from initiation of donor searching to transplant can be significant, averaging around 3 months.

In conclusion, our results show that MA haplo-HSCT results in favorable engraftment, acceptable rates of GVHD, and low nonrelapse mortality. Relapse rates appear lower than that reported with NMA haplo-HSCT. DRI represents the strongest predictor of outcome following MA haplo-HSCT and PT/Cy. Disease-free and overall survival is equivalent to recipients of MA MUD transplants. Therefore, in younger patients without contraindications to standard intensity conditioning, MA haplo-HSCT is a valid option for patients with advanced hematologic malignancies who lack timely access to a conventional donor.

## Figures and Tables

**Table 1:** Predictors of transplant outcomes following MA haplo HSCT.

	OS		DFS		NRM		Relapse	
	HR	*p*	HR	*p*	HR	*p*	HR	*p*
DRI (high versus low/int)	**13.26**	*<0.001*	**10.84**	*<0.001*	**15.0**	*0.004*	**8.85**	*0.004*
CMI (≥2 versus <2)	**3.54**	*0.010*	**3.09**	*0.018*	**13.6**	*0.007*	—	—
Age (<50 versus ≥50)	**1.26**	*0.038*	**1.31**	*0.015*	**1.43**	*0.055*	—	—

The following variables were considered in Cox analysis: age, diagnosis, Karnofsky performance status (KPS), comorbidity index (CMI), revised Dana-Farber disease risk index (DRI), conditioning regimen (busulfan versus TBI), year of transplant, acute GVHD, and chronic GVHD. Variables were selected by 10% threshold. Acute and chronic GVHD were modeled as time-dependent variables.
